# Neonatal myoclonus in Bryant-Li-Bhoj syndrome associated with a novel *H3F3A* variant

**DOI:** 10.1038/s41439-024-00303-x

**Published:** 2024-12-04

**Authors:** Moemi Hojo, Noriko Soma, Kei Yamada, Yu Kobayashi, Masaki Miura, Hitomi Fujii, Hiromi Nyuzuki, Yosuke Nishio, Taichi Oso, Tomoo Ogi, Takeshi Ikeuchi, Jun Tohyama

**Affiliations:** 1 Department of Child Neurology, National Hospital Organization Nishiniigata Chuo Hospital, Niigata, Japan; 2 Center for Medical Genetics, Niigata University Medical and Dental Hospital, Niigata, Japan; 3 Department of Pediatrics, Nagoya University Graduate School of Medicine, Nagoya, Japan; 4 Department of Genetics, Research Institute of Environmental Medicine (RIeM), Nagoya University, Nagoya, Japan; 5 Department of Human Genetics and Molecular Biology, Nagoya University Graduate School of Medicine, Nagoya, Japan

**Keywords:** Disease genetics, Medical genetics

## Abstract

Bryant-Li-Bhoj syndrome (BLBS; OMIM # 619720, 619721), caused by germline *H3F3A* and *H3F3B* variants encoding histone H3.3, is characterized by mild to severe developmental delay, intellectual disability, failure to thrive, muscle tone abnormalities, and dysmorphic facial features. Here, we present a Japanese patient with a novel heterozygous p.A48G variant in *H3F3A*, displaying previously unrecognized symptoms of neonatal myoclonus. This case helps broaden the phenotypic spectrum of BLBS.

Somatic variants of *H3F3A* and *H3F3B*, which encode histone H3.3, are known to cause brain or bone tumors^1,2^. Recently, germline variants in these two genes have been reported to cause specific neurodevelopmental and neurodegenerative disorders, such as Bryant-Li-Bhoj syndrome^3^ (BLBS; OMIM # 619720, 619721). The first patient with BLBS was reported in 2019^4^, and to date, nearly 100 cases have been reported^3–7^. This syndrome is characterized by mild to severe developmental delay, intellectual disability, failure to thrive, microcephaly, muscle tone abnormality, and dysmorphic facial features. Approximately half of the patients with this syndrome experience epilepsy, most often during infancy or childhood^8^.

Herein, we report the case of a Japanese patient who presented with myoclonus during the neonatal period, profound intellectual disability, seizures, and a de novo heterozygous *H3F3A* variant. To the best of our knowledge, this is the first report of a Japanese patient with BLBS, providing detailed clinical findings that contribute to expanding the phenotypic spectrum of BLBS.

A female patient, the third child of unrelated parents, was born at 36 weeks gestation after a normal pregnancy and delivery. Her birth weight was 1958 g (−1.9 standard deviation score [SDS]), length was 43.5 cm (−1.7 SDS), and head circumference was 29.0 cm (−2.6 SDS). The patient’s family history was unremarkable. At two days of age, she developed myoclonic movements in both extremities, which gradually increased in frequency despite phenobarbital administration. She was admitted to our hospital at 21 days of age. The anthropometric measurements at one month of age were as follows: body weight, 2.66 kg (−2.8 SDS); height, 45.5 cm (−3.4 SDS); and head circumference, 33.0 cm (−2.2 SDS). The findings of the physical examination were normal. Frequent myoclonic movements were observed after admission. Routine laboratory tests, including screening for metabolic disorders and chromosomal karyotypes and brain magnetic resonance imaging (MRI), were normal. An interictal electroencephalogram (EEG) revealed no spike discharges with a normal background and no EEG abnormalities corresponding to myoclonic movements. Therefore, it was not clear whether her myoclonic movements were epileptic or nonepileptic in origin. However, after the administration of valproic acid, her myoclonus gradually decreased. At 2 years of age, she developed generalized tonic-clonic seizures during the febrile state and later experienced focal impaired awareness seizures at 3 years and 5 months and again at 4 years and 3 months of age. Thereafter, myoclonus and focal seizures were no longer observed, and valproic acid was discontinued at 8 years of age. However, her developmental delays became progressively noticeable, and her intelligence quotient at 6 years of age was 42 on the Suzuki–Binet test. Her facial dysmorphic features gradually became evident (Fig. 1a), and she had to undergo surgery for strabismus at 2 years of age.

**Fig. 1:**
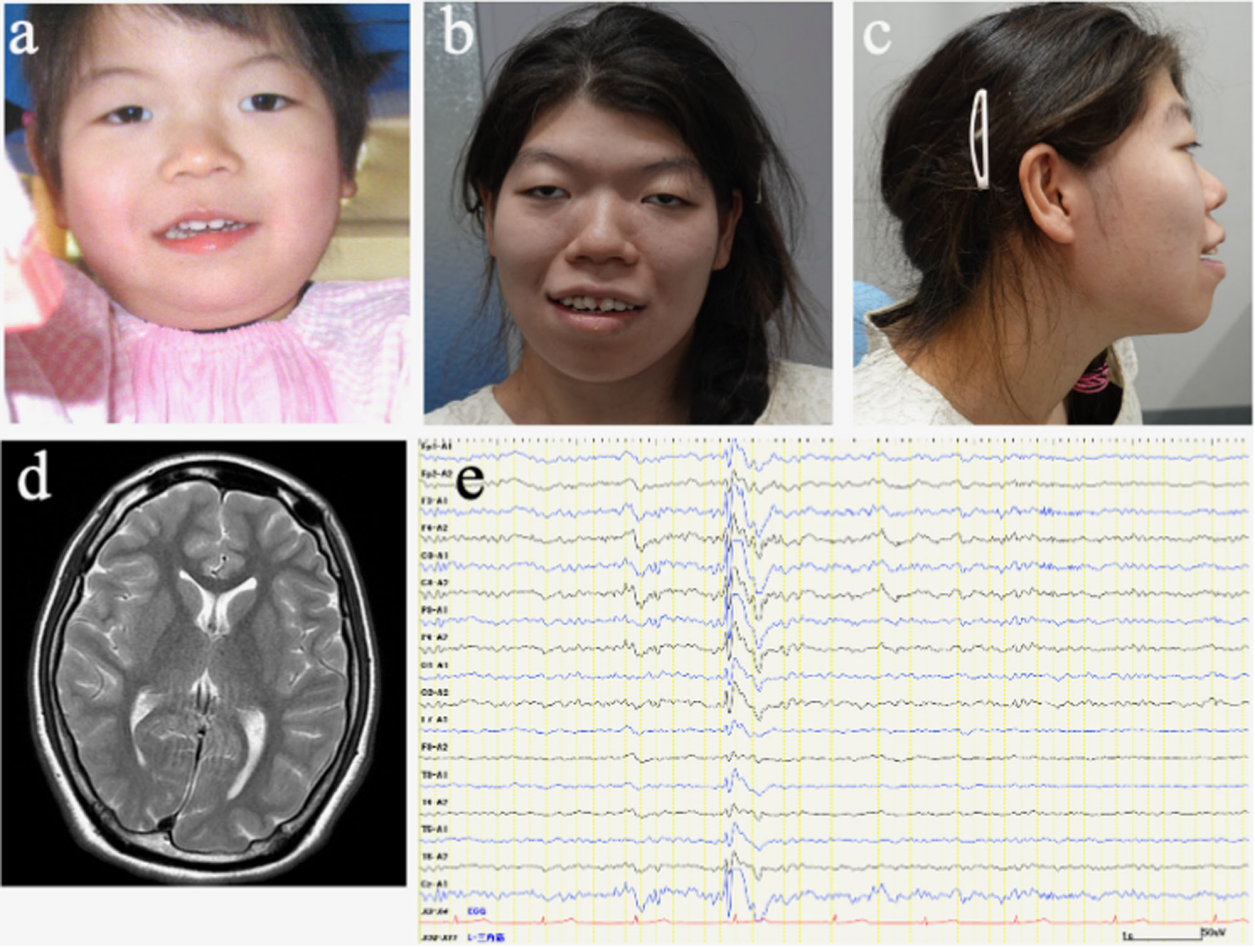
Clinical, neuroradiological and neurophysiological features of the patient. **a** The facial features of the patient at 3 years and 6 months of age include hypertelorism, strabismus, a wide nasal bridge and a long philtrum. **b**, **c** Facial features of the patient at 18 years of age include hypertelorism, strabismus, a wide and depressed nasal bridge, and thick lips. **d** Brain magnetic resonance imaging at 18 years of age. T2-weighted axial images show normal findings. **e** Interictal electroencephalogram at 18 years of age showing spike waves in the bilateral frontal, central and parietal regions during sleep.

At 18 years and 9 months of age, she experienced focal motor seizures again, which evolved into bilateral tonic-clonic seizures, and was admitted to our hospital. Her body weight was 43.5 kg (−1.2 SDS), and her height was 147.8 cm (−1.9 SDS). She exhibited specific facial features, such as hypertelorism, strabismus, a wide and depressed nasal bridge, and thick lips (Fig. 1b, c). No abnormalities in muscle tone or involuntary movements were observed. The brain MRI findings were normal (Fig. 1d). Interictal EEG revealed spike discharges or spike-and-wave discharges in both the frontal and parietal areas (Fig. 1e). Two months later, she developed another generalized tonic-clonic seizure, and she was administered lacosamide. However, it was ineffective, and her seizures resolved with the administration of levetiracetam. Her development was severely delayed, with an intelligence quotient of 20 on the Tanaka–Binet test at 18 years of age. She could walk independently and speak simple words. She occasionally exhibited impulsive behavior and received risperidone.

At 18 years of age, genomic DNA was collected from the patient and her parents for whole-exome analysis. Consequently, a de novo c.143 C > G (p.Ala48Gly) variant was identified in *H3F3A* (NM_002107). This novel variant was confirmed through Sanger sequencing. The variant is classified as likely pathogenic, according to the American College of Medical Genetics and Genomics-Association of Molecular Pathology (ACMG-AMP) Guidelines (PS2 and PM2). Although some in silico tools support pathogenicity, such as combined annotation-dependent depletion with a Phred score of 28.5, the conflicting predictions from other tools have led us to conservatively refrain from applying PP3. Clinical and molecular genetic studies were performed in accordance with the Declaration of Helsinki and approved by the Institutional Review Board of Niigata University School of Medicine. This study was supported by the Japanese Agency for Medical Research and Development (AMED) through the Initiative on Rare and Undiagnosed Diseases (IRUD). Written informed consent was obtained from the parents of the patient.

The patient exhibited severe intellectual disability, seizures, and specific facial features, and a diagnosis of BLBS could be made after comprehensive genetic testing. We compared the clinical features of our patient with those of a previously reported summary of BLBS^3^ (Table 1). Our patient presented many characteristic symptoms, including a dysmorphological facial appearance similar to that of BLBS patients; however, she had no musculoskeletal, dermal, or genital anomalies. Notably, genital anomalies are more commonly reported in male patients. Almost half of the previously reported patients had musculoskeletal anomalies and dermal symptoms of BLBS^3^. The absence of these symptoms in our patients may have been due to phenotypic variability.

**Table 1:** Phenotypic manifestations of the present patient with the p.A48G variant and summary of previously reported cases.

	Summery by Layo-Carris et al.^3^	This case
Demographics		
Age	2 months - 39 years	20 years
Sex	Males/Females = 47/49	female
Growth		
Height (>95th percentile)	6/91 (7%)	-
Height (≤5th percentile)	32/91 (35%)	-
Weight (>95th percemtile)	14/76 (18%)	-
Weight (≤5th percemtile)	15/76 (20%)	-
Macrocephaly (≥95th percentile)	14/95 (15%)	-
Microcephaly (≤５th percentile)	30/95 (32%)	+
Craniofacial anomalies	86/93 (92%)	+
Neuroimaging findings	44/76 (58%)	-
Neurodevelopment		
Developmental delay/intellectual disability	94/95 (99%)	+
Seizures	45/91 (49%)	+
Delayed/No sitting (>12 months)	33/65 (51%)	-
Delayed/No walking (>20 months)	59/75 (79%)	-
Speaks at least one word (>20 months)	50/84 (60%)	-
Muscle tone abnomalies		
Hypotonia	57/92 (62%)	-
Hypertonia	11/92 (12%)	-
Axial hypotonia, peripheral hypertonia	9/92 (10%)	-
Oculomotor	49/90 (54%)	+
Strabismus	32/90 (36%)	+
Astigmatism	7/88 (8%)	-
Review of systems		
Musculoskeletal	56/94 (60%)	-
Cardiac	11/82 (13%)	-
Dermal	46/88 (52%)	-
Genital	17/85 (20%)	-

We assumed that the severity of the phenotype did not correlate with the location of the variant. The H3.3 protein comprises a disordered tail, four α-helices, and two loop domains. *H3F3A* variants are associated with either the tail domain or core domain. Layo-Carries et al. investigated whether the variants in these two domains were associated with the clinical symptoms of BLBS; however, specific phenotype-genotype correlations were not delineated. While the p.A48G variant of the present patient was also located in the core domains, she did not exhibit all the symptoms commonly observed in the core domain variants^3^. Typically, many BLBS patients do not achieve independent sitting, walking or speaking^8^. Although the patient had a profound intellectual disability, she was able to walk without support and speak simple words, suggesting that her condition may have been relatively mild. These phenotypic variations may be due to either modifying alleles or environmental factors^5^.

In the present patient, myoclonic movement in the neonatal period was a hallmark symptom. As video-EEG monitoring did not confirm whether the myoclonic movement was an epileptic seizure, we considered that the myoclonus in the present patient was involuntary movement. Myoclonus can originate at multiple levels of neuraxis and can occur in numerous neurological conditions^9^. The pathophysiology of myoclonus involves a brief inappropriate discharge of neurons that are transmitted to the body, resulting in a brief jerk. Therefore, increased neural excitability is presumed to be observed in some regions in patients with BLBS. Although the pathophysiology or origin of the myoclonus in the present patient is unknown, BLBS may be a neurodevelopmental condition that results in myoclonus.

Approximately half of individuals with BLBS experience epilepsy or seizures^8^. The type of seizure is variable and includes myoclonic, tonic, focal and tonic-clonic seizures^8^. The onset of epilepsy generally occurs during infancy or childhood. Hu et al. investigated 470 patients with genetic early infantile-onset developmental and epileptic encephalopathy using next-generation sequencing and identified the *H3F3A* variant in one patient with non-syndromic developmental and epileptic encephalopathy^7^. Our patient had myoclonus in the neonatal period, followed by recurrent focal seizures and tonic-clonic seizures since the age of two years. Her seizures were infrequent and well controlled with valproic acid or levetiracetam. Therefore, the epilepsy in the present patient did not fit the conditions of developmental and epileptic encephalopathy.

Many symptoms in the present case correspond to the symptoms of BLBS, recognized as a neurodevelopmental syndrome with a characteristic craniofacial appearance. The germline variant in the present patient was located at a different site from the hotspot tumor-causing somatic variants, which is also consistent with previous results^3^. This is the first report of a Japanese patient with BLBS, which helps broaden the phenotypic spectrum of BLBS.

## Data Availability

The relevant data from this Data Report are hosted at the Human Genome Variation Database at 10.6084/m9.figshare.hgv.3468.
